# Distribution of Human Papillomavirus Genotypes in Real‐World Cervical Self‐Collected Scrapings From the Dutch Cervical Cancer Screening Program

**DOI:** 10.1002/jmv.70461

**Published:** 2025-07-02

**Authors:** Kelly Melisa Castañeda, Jolien de Waard, Lorian Slagter‐Menkema, Mirjam Mastik, Floris Adriaan Vuijk, Rudolf Lambertus Maria Bekkers, Geertruida Hendrika de Bock, Gijsbertha Barendina Alida Wisman, Ed Schuuring

**Affiliations:** ^1^ Department of Epidemiology University of Groningen, University Medical Center Groningen Groningen the Netherlands; ^2^ Department of Pathology & Medical Biology University of Groningen, University Medical Center Groningen Groningen the Netherlands; ^3^ Department of Gynecologic Oncology, Cancer Research Center Groningen University of Groningen, University Medical Centre Groningen Groningen the Netherlands; ^4^ Department of Otorhinolaryngology University of Groningen, University Medical Center Groningen Groningen the Netherlands; ^5^ MSD B.V. Haarlem the Netherlands; ^6^ Department of Obstetrics and Gynecology Radboud University Medical Center, Nijmegen Nijmegen the Netherlands; ^7^ Department of Obstetrics and Gynecology Catharina Hospital Eindhoven the Netherlands; ^8^ GROW School forty Oncology and Reproduction Maastricht University Maastricht the Netherlands

**Keywords:** human papillomavirus viruses, prevalence, uterine cervical neoplasms

## Abstract

High‐risk HPV (hrHPV) is the necessary cause of cervical cancer with HPV16/18 accounting for around 70% of the cases worldwide, while other non‐HPV16/18 hrHPV genotypes prevail in ~95% of high‐grade lesions. Understanding regional genotype distribution of hrHPV types not covered by the nonavalent vaccine is crucial for evaluating vaccine effectiveness and enhancing population‐based screening (PBS). The objective of the present study is to update hrHPV genotype prevalence in a non‐vaccinated cohort of 1200 hrHPV‐positive women from the Dutch PBS using INNO‐LiPA HPV Genotyping Extra‐II to identify 32 individual HPV genotypes in self‐sampled material. HrHPV prevalence for all 32 genotypes, also grouped by bivalent, quadrivalent, and nonavalent vaccine types (2vHPV, 4vHPV, and 9vHPV), was reported by histologic diagnosis and age. The most common genotypes were HPV16 (394,33%), especially in younger women, followed by HPV31 (216,18%) and HPV52 (199,17%). 2vHPV genotypes were found in 23% (*n* = 90) of NILM cases, 27% (*n* = 84) of CIN0/CIN1, 45% (*n* = 74) of CIN2, 71% (*n* = 219) of CIN3, and 92% (*n* = 12) of cervical cancers. In comparison, 9vHPV genotypes appeared in 60% (*n* = 240) of NILM, 69% (*n* = 218) of CIN0/CIN1, 88% (*n* = 145) of CIN2, 94% (*n* = 289) of CIN3, and all cervical cancers (*n* = 13). HrHPV types not included in 9vHPV had an overall prevalence of 19% (*n* = 225), with 88% (197/225) found in NILM or CIN0/CIN1. This study highlights vaccine‐type HPV in all cancer cases and many high‐grade lesions, reinforcing the need for improved vaccination efforts and broader protection.

**Abbreviations**
2vHPV: bivalent HPV vaccine
4vHPV: quadrivalent HPV vaccine
9vHPV: nonavalent HPV vaccine
CI: confidence intervals
CIN: cervical intraepithelial neoplasia
HPV: human papillomavirus
hr: high‐risk
IARC: International Agency for Research on Cancer
lr: low‐risk
NILM: negative for intraepithelial lesion or malignancy
PALGA: Dutch Nationwide Pathology Databank
PBS: population‐based screening

## 1 Introduction

Cervical cancer is the fourth most common cancer in women worldwide, with human papillomavirus (HPV) infection being the main risk factor [1]. HPVs can be classified as high‐ or low‐risk based on their oncogenic potential [2]. High‐risk HPV (hrHPV) is the necessary cause of cervical cancer, with around 70% of all cervical cancers being attributable to hrHPV types 16 or 18, and 96% to one of the 13 hrHPV types (HPV16, 18, 31, 33, 35, 39, 45, 51, 52, 56, 58, 59, and 68) [3]. Meanwhile, low‐risk HPV (lrHPV) is usually associated with anogenital warts and recurrent respiratory papillomatosis, with types 6 and 11 known to cause approximately 90% of these papillomas [4, 5].

The prevalence of HPV16 and 18 within cervical cancer is relatively consistent worldwide, while the distribution of other HPV genotypes showed slight variations between geographical regions and precancerous lesions [6]. Because current cervical screening focuses on the early detection of high‐grade lesions rather than cancer only, in addition to HPV16/18, the other hrHPV genotypes are tested routinely to select women at high risk of disease (high‐grade lesions and cervical cancer) [7]. The most commonly used clinical hrHPV assays for early detection cover the hrHPV types associated with cervical cancer (with lower analytical sensitivity), mostly providing no information on the presence of hrHPV genotypes or co‐infections, separately [7]. Studies evaluating the prevalence of HPV genotypes across different histological stages, negative for intraepithelial lesion or malignancy (NILM), cervical intraepithelial neoplasia grades 1 (CIN1), 2 (CIN2), 3 (CIN3) and cervical cancer, have highlighted several key findings: (1) a relatively high prevalence of HPV not only in cancer but also in high‐grade lesions and normal samples [8], (2) frequent co‐infections, particularly in earlier stages [9], and (3) significant regional differences in HPV genotype distribution [6].

Since 2006, following the approval of prophylactic HPV vaccines by the Food and Drug Administration and the European Medicines Agency, the primary strategy for the prevention of HPV infections and related diseases has been the use of bivalent, quadrivalent, or nonavalent vaccines (2vHPV, 4vHPV, 9vHPV). The 2vHPV vaccine targets types HPV16 and 18, the 4vHPV vaccine targets types HPV6, 11, 16, and 18, while the 9vHPV vaccine targets types HPV31, 33, 45, 52, and 58, in addition to the aforementioned types (HPV6, 11, 16, and 18) [10]. Now, with the implementation of HPV vaccines, understanding the distribution of various (combinations of) hrHPV genotypes by region becomes crucial to evaluate the impact of HPV vaccines and enhance better management of women within HPV‐based cervical cancer screening programs in specific sites [11, 12].

In the Netherlands, data on the distribution of individual HPV genotypes is relatively old (1999–2002) and limited to the Amsterdam region [13]. Since the start of the national HPV vaccination program in 2010 with 2vHPV, the HPV genotype distribution may have changed. Therefore, the aim of this study is to provide an up‐to‐date overview of the prevalence of various HPV genotypes in hrHPV‐positive non‐vaccinated women from the Dutch population‐based screening (PBS) program. For this purpose, we used a test capable of detecting 32 individual HPV genotypes with high analytical sensitivity.

## 2 Materials and Methods

### 2.1 Patient Samples and Definitions

To ensure the inclusion of a population‐based series of non‐vaccinated women, we selected women aged 30–63 years who participated in the national PBS program in the North of the Netherlands between July 2020 and March 2022, as the first group of vaccinated women would not be eligible to participate until 2023. Their samples were obtained using a self‐sampling device at home (Evalyn Brush, Rovers Medical Devices B.V., Oss, the Netherlands), and after transporting to the central laboratory at UMCG resuspended and preserved in 20 mL of ThinPrep medium (Hologic Inc., Marlborough, MA, USA). All samples in this study tested positive for hrHPV using the Cobas 4800 clinical hrHPV test (Roche Diagnostics, Alameda CA, USA) within the regular PBS. Self‐collected samples were stored at room temperature at the UMCG and were processed for DNA extraction and genotyping between August 2023 and February 2024. Histopathological classification of the tissue/biopsies taken by the gynaecologist during colposcopy was used as the gold standard for defining disease stage. Histology and cytology results were retrieved from the the Dutch Nationwide Pathology Databank (PALGA). Histology was categorized as CIN0, CIN1, CIN2, CIN3 and cancer. Women with two consecutive normal cytology results (at primary screening [*t* = 0 months] and ~6 months of follow‐up screening [control smear], and therefore not referred to the gynaecologist for colposcopy) were considered as hrHPV‐positive women without disease (i.e., NILM).

### 2.2 DNA Extraction

DNA extraction was performed on the same samples used for routine hrHPV testing in the PBS program. The Promega Maxwell RSC Viral Total Nucleic Acid Kit (Promega Corporation, Madison, WI, USA) was used according to the manufacturer protocol. The ThinPrep vial was vortexed briefly to resuspend cells, following which 300 µL of the sample was transferred to a 1.5 mL microtube. Subsequently, 300 µL of the lysis buffer and 30 µL of proteinase K were added to the sample and vortexed for 10 s. The sample was then incubated at 56°C for 10 min. Following the sample incubation, the entire volume of lysate was transferred to the Maxwell RSC cartridge and processed in the Maxwell RSC Instrument using the Viral Total Nucleic Acid method. The isolated DNA was then eluted in 50 µL Nuclease‐Free Water [14]. For quality control of the extracted DNA, DNA from the first 100 samples were also amplified using the BIOMED‐2 PCR protocol [15]. All 100 samples contained sufficient amplified DNA and revealed PCR‐products of at least 400 bp indicating high quality of DNA. Because all the included samples also showed sufficient DNA in the Cobas 4800 clinical hrHPV test, we assumed that the rest of the samples would also have sufficient good‐quality DNA. The INNO‐LiPA HPV Genotyping Extra II assay (see next paragraph) also includes an internal control for sufficient DNA input and all samples were valid.

### 2.3 HPV Genotyping

The INNO‐LiPA HPV Genotyping Extra II assay (Innogenetics N.V., Ghent, Belgium) was utilized to identify 32 different HPV genotypes individually (HPV16, 18, 31, 33, 35, 39, 45, 51, 52, 56, 58, 59, 68, 26, 53, 66, 70, 73, 82, 6, 11, 40, 42, 43, 44, 54, 61, 81, 62, 67, 83, and 89), providing individual results for each genotype, and ensuring high analytical sensitivity. These analyses were conducted following the instructions of the manufacturer. This assay involves an initial PCR amplification step, followed by a line probe assay based on reverse hybridization [16]. In short, 10 µL of the isolated DNA was subjected to a short PCR fragment assay using biotinylated consensus primers, amplifying a 65 bp fragment in the conserved L1 region of multiple alpha HPV types [16]. Additionally, a primer set targeting the human HLA‐DPB1 gene was included to assess the quality of extracted DNA. To identify the HPV type‐specific sequences, the resulting biotinylated amplicons were denatured and hybridized to filter‐strips each containing various HPV‐specific oligonucleotide probes. The INNO‐LiPA assay, including sample incubation, stringent wash, and color development, was performed using the TENDIGO system according to manufacturer's protocol (INNO‐LiPA; Fujirebio.com). The results of each sample were independently scored manually by two researchers using the INNO‐LiPA HPV Genotyping Extra II Reading Card. Intensely colored line probes were scored as 1, while those faintly colored but still visible to the naked eye and after capturing a picture were scored as 2. A score of 0 was assigned when there was no evidence of color after capturing the pictures. For this study, scores of 1 and 2 were considered positive for the specific genotype line probe, while score 0 was considered negative. If multiple infections were present within the same sample, we assessed each genotype individually. Samples that did not show positive results for any of the 32 genotype line probes were classified as HPV‐negative according to the INNO‐LiPA test. Contamination of amplification products was prevented by all standard precautions, using separate laboratories for pre‐ and post‐PCR handling in a NEN‐ISO15189 accredited laboratory.

### 2.4 Statistical Analysis

All statistical analyses were conducted using R version 4.3.1, with the following packages: gtsummary, ggplot2, ComplexUpset, binom, and nnet. Firstly, the prevalence of all 32 HPV genotypes was computed for each histologic diagnosis, cytology results, and age group. The prevalence of each genotype was calculated both when it appeared alone (single infection) and when it appeared with other types (multiple infections) in the total population. This was done overall, as well as for CIN2+ (CIN2, CIN3, and cancer) and CIN3+ (CIN3 and cancer). The results were plotted in a stacked bar plot. This plot shows the segments representing single and multiple infections stacked on top of each other. The total length of the bar represents the total prevalence. To visualize the most common patterns (either single or multiple) of HPV infection in our population, we applied upset plots (a plot representing a unique pattern or combination of categories, in this case HPVs, and the length of the bar indicating the prevalence of the specific combination). Secondly, we explored the prevalence of HPV using three different classifications: (1) HPV vaccine types (HPV16/18 [2vHPV], HPV6/11/16/18 [4vHPV], and HPV6/11/16/18/31/33/45/52/58 [9vHPV]). (2) Based on the cancer risk classification established by the International Agency for Research on Cancer (IARC) [11], we employed a hierarchical system where groups were mutually exclusive, with each participant assigned to only one group. HrHPV genotypes were categorized into five risk‐based groups: (a) HPV16, (b) HPV18/45, (c) HPV33/31/52/58/35, (d) HPV39/51/59/56/68, and (e) negative for hrHPV. (3) We used the number of hrHPV types within each sample, categorizing participants as positive for one, two or three and/or more hrHPV genotypes. The prevalence of each classification per histological diagnosis and age group was estimated with 95% confidence intervals (95% CI), using the normal approximation of the binomial distribution. Finally, to evaluate the relationship between high‐risk genotypes included in the 9vHPV vaccine, age, and histologic diagnosis, and to determine if age distribution influences the association between hrHPV of the 9vHPV and histologic diagnosis, we performed univariable and multivariable logistic regression analyses. Age and histologic diagnosis were considered independent variables, while the presence of high‐risk genotypes included in the 9vHPV (16/18/31/33/45/52/58) was the dependent variable.

## 3 Results

### 3.1 Study Population and Overall HPV Genotype Prevalence

In total, 1200 hrHPV‐positive women from the Dutch cervical cancer screening program were included in our population‐based series for HPV genotyping. Among them, 400 cases were classified as NILM, 315 as CIN0/1, 164 as CIN2, 308 as CIN3, and 13 as cervical cancer. 56% of the total population were between 30 and 39 years old, and most CIN3+ cases (51%) occurred within this age group (Supporting Information S1: Table 1).

**Figure 1:**
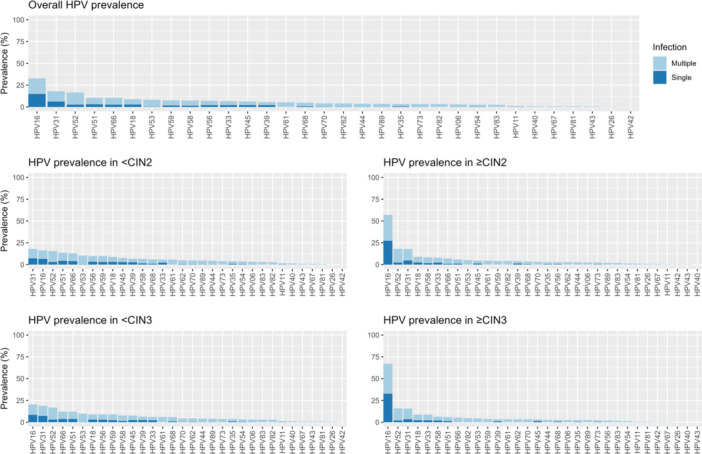
Stacked bar plot showing the overall prevalence of HPV genotypes as well as the prevalence for CIN2+ and CIN3+, categorized by single (dark blue) and multiple (light blue) infections. Each bar in the plot represents a specific HPV genotype, with the total height corresponding to its overall prevalence. Within each bar, segments are divided to represent the prevalence of the genotype when occurring alone (single infection) in the dark blue and when co‐occurring with other genotypes (multiple infections) in light blue. HPV types are ranked by frequency.

**Figure 2:**
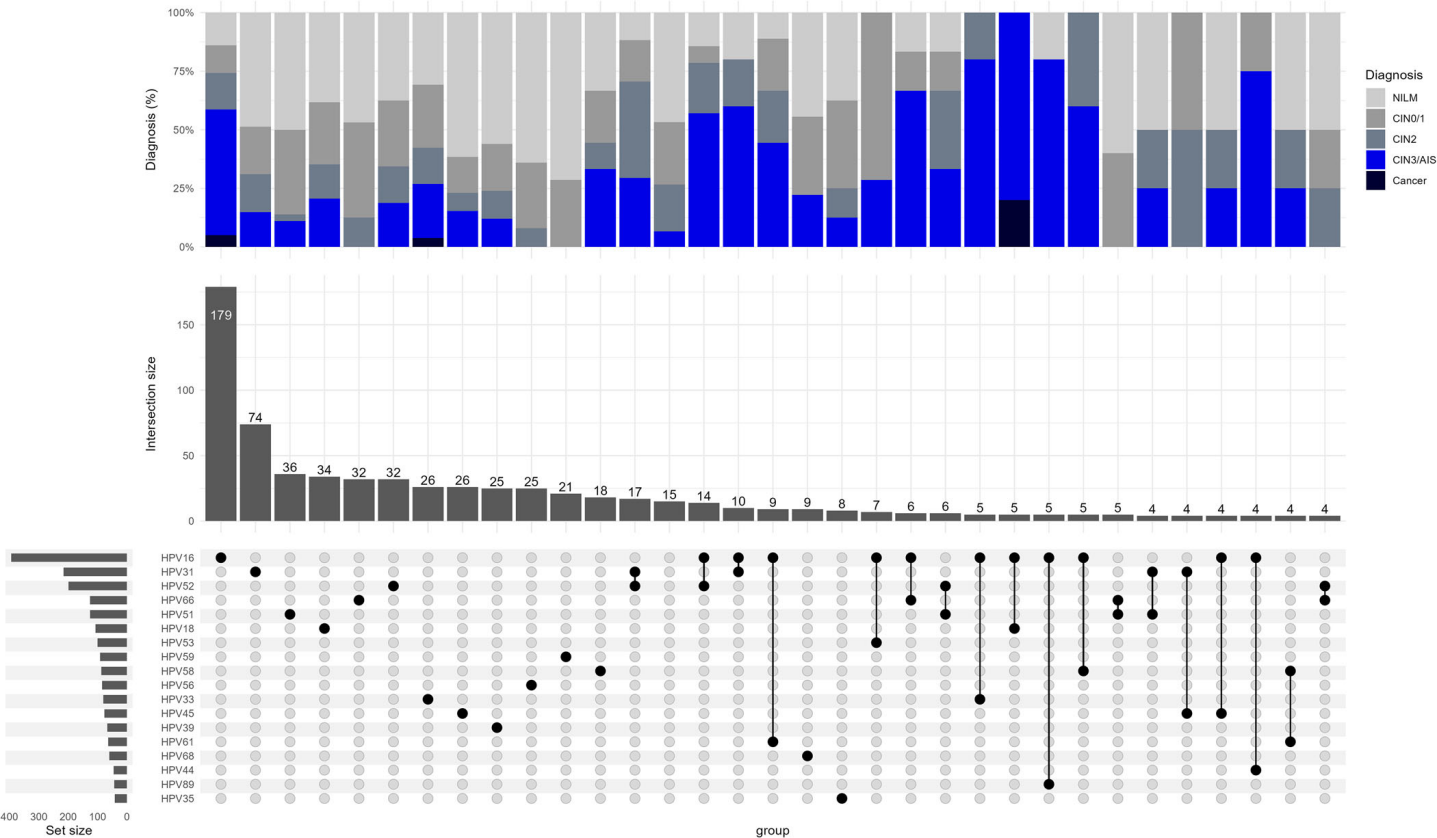
Upset plot showing the frequencies of HPV genotypes (single and multiple infections) in set size, and the pattern of HPV infections in intersection size. Each dot in the matrix represents positivity for the respective HPV in the row. When two dots are connected by a line, it denotes a coinfection. The stacked bars at the top indicate the percentages of histologic diagnosis based on the patterns of HPV infections. Only patterns of HPV infections occurring in four or more participants are presented. Ranking is based on frequency.

In the total cohort, at least one of any 32 HPV genotypes was identified in 1185 out of the 1200 samples (98.8%). The top three most prevalent HPV genotypes were HPV16 (33%), HPV31 (18%), and HPV52 (17%) (Figure 1). 52% (624/1200) of the samples had multiple infections (considering all 32 genotypes evaluated). The most prevalent pattern of HPV infection was a single infection with HPV16 (*n* = 180), followed by a single infection with HPV52 (*n* = 74), and a single infection with HPV51 (*n* = 36) (Figure 2). Although half of the samples had co‐infections, the frequency of a specific combination of HPVs was relatively low. The combination of HPV31 and HPV52 was the most frequent co‐infection, observed in 17 participants, whereas only 5 participants had co‐infections of HPV16 and HPV31, and only 4 participants had co‐infections of HPV52 and HPV66 (Figure 2).

### 3.2 Prevalence of HPV Genotypes by Histology Diagnosis

The prevalence of HPV16 increased with the severity of the underlying lesion. Although HPV31 was the second most common type, its prevalence was relatively high in high‐grade lesions but low in cancer cases (only one case (out of 13) was positive for HPV31, and it was in a co‐infection with HPV18) (Supporting Information S1: Table 2). The prevalence of HPV16 was 57% in CIN2+ cases and 33% in < CIN2 cases. Similarly, HPV16 prevalence was 67% in CIN3+ cases and 20% in < CIN3 cases. Approximately 60% of HPV16 infections in both CIN2+ and CIN3+ cases were single infections. Most of the low‐risk genotypes were presented as multiple infections only (Figure 1).

### 3.3 Distribution of hrHPV by Different Classifications

When using the IARC classification, we observed that 1116 (93%) of the total samples tested positive for hrHPV. Of the 84 (7%) hrHPV‐negative samples, four of them were CIN3 (1.3%, 95% CI: 0.42–3.5). HPV16 was present in 66% (95% CI: 61–71) of CIN3 cases and 85% (95% CI: 54–97) of cancer cases. Additionally, we observed an increasing prevalence of HPV16 as the severity of the lesions increased. Conversely, there was a decreasing tendency in the prevalence of HPV59/39/68/51/56 with the increasing severity of the lesions (*p* < 0.001) (Table 1).

**Table 1:** Prevalence of high‐risk HPV based on HPV vaccine types, IARC Classification, and number of high‐risk HPV infections per histology diagnosis.

HPV	Overall *N* = 1200 *n* (%, 95% IC)	NILM *N* = 400 *n* (%, 95% IC)	CIN0/1 *N* = 315 *n* (%, 95%IC)	CIN2 *N* = 164 *n* (%, 95% IC)	CIN3/AIS *N* = 308 *n* (%, 95% IC)	Cancer *N* = 13 *n* (%, 95% IC)
HPV vaccine types						
2vHPV	479 (40%, (37–43)	90 (23%, 19–27)	84 (27%, 22–32)	74 (45%, 37–53)	219 (71%, 66–76)	12 (92%, 62–100)
4vHPV	507 (42%, 39–45)	98 (25%, 20–29)	99 (31%, 26–37)	76 (46%, 39–54)	222 (72%, 67–77)	12 (92%, 62–100)
9vHPV	905 (75%, 73–78)	240 (60% 55–65,)	218 (69%, 64–74)	145 (88%, 82–93)	289 (94%, 90–96)	13 (100%,72–100)
IARC classification						
HPV16	394 (33%, 30–36)	58 (15%,11–18)	59 (19%, 15–24)	62 (38%, 30–46)	204 (66%, 61–71)	11 (85%, 54–97)
HPV18/45	150 (13%, 11–15)	61 (15%, 12–19)	45 (14%, 11–19)	22 (13%, 8.8–20)	21 (6.8%, 4.4–10)	1 (7.7%, 0.40–38)
HPV33/58/31/52/35	360 (30%, 27–33)	122 (31%, 26–35)	110 (35%, 30–41)	62 (38%, 30–46)	65 (21%, 17–26)	1 (7.7%, 0.40–38)
HPV59/39/68/51/56	212 (18%,16–20)	115 (29%, 24–34)	73 (23%, 19‐28)	10 (6.1%, 3.1–11)	14 (4.5%, 2.6–7.7)	0 (0%)
Negative for hrHPV	84 (7%, 5.7–8.6)	44 (11%, 8.2–15)	28 (8.9%, 6.1–13)	8 (4.9%, 2.3–9.7)	4 (1.3%, 0.42–3.5)	0 (0%)
Number of hrHPV types						
0	84 (7%, 5.7–8.6)	44 (11%, 8.2–15)	28 (8.9%, 6.1–13)	8 (4.9%, 2.3–9.7)	4 (1.3%, 0.42–3.5)	0 (0%)
1	759 (63%, 60–66)	274 (69%, 64–73)	181 (57%, 52–63)	93 (57%, 49–64)	201 (65%, 60–71)	10 (77%, 46–94)
2	236 (20%, 17–22)	52 (13%, 9.9–17)	69 (22%, 18–27)	44 (27%, 20–34)	69 (22%, 18–28)	2 (15%, 2.7–46)
≥ 3	121 (10%, 8.5–12)	30 (7.5%, 5.2–11)	37 (12%, 8.5–16)	19 (12%, 7.3–18)	34 (11%, 7.9–15)	1 (7.7%, 0.40–38)

With the number of hrHPV genotypes, we observed that overall, 63% (95% CI: 60–66) of the samples had only one hrHPV genotype, and around 30% of the samples had two or more hrHPV types. The distribution of the number of hrHPV genotypes per histologic diagnosis seems quite consistent (Table 1).

Using the hrHPV vaccine types classification, 40% (479/1200) of the samples were positive for HPV types present in the 2vHPV, with 64% (305/479) of these cases classified as CIN2+ and 48% (231/479) as CIN3+. 42% (507/1200) of the samples were positive for HPV types present in the 4vHPV, showing similar distribution of cases. Additionally, 75% (905/1200) of the samples had HPV types present in the 9vHPV, with 49% (447/905) classified as CIN2+ and 33% (302/905) as CIN3+. In other words, HPV16/18 (2vHPV) is present in 23% of NILM, 27% of CIN0/CIN1, 45% of CIN2, 71% of CIN3, and 92% of cervical cancer cases. When including all HPV types covered by the 4vHPV (HPV6/11/16/18), these percentages remain the same as for the 2vHPV (25% for NILM, 31% for CIN0/CIN1, 46% for CIN2, 72% for CIN3, and 92% for cervical cancer). When considering all types included in the 9vHPV (HPV6/11/16/18/31/33/45/52/58), these percentages increase to 60% for NILM, 69% for CIN0/1, 88% for CIN2, 94% for CIN3, and 100% for cancer cases (Table 1).

### 3.4 Prevalence of HPV Genotypes by Age

The prevalence of HPV16 and HPV31 decreased with age, while other genotypes, such as HPV18, decreased from ages 30 to 40, then increased, and decreased again after age 50 (Supporting Information S1: Table 3). The percentage of women aged 30‐34 was higher among those with an HPV16 infection, regardless of whether the infection was single or a coinfection (Supporting Information S1: Figure 1).

Analyzing hrHPV prevalence using the IARC classification, we observed that the prevalence of other hrHPV genotypes different than HPV16 either increased or remained stable with increasing age (Table 2). Additionally, the proportion of individuals negative for hrHPV genotypes (0 hrHPV) increased with age, while the presence of one or two hrHPV genotypes remained relatively stable throughout life. In contrast, the occurrence of three or more high‐risk genotypes decreased from ages 30 to 49 but increased again starting at age 50 (Table 2). Using the classification of all vaccine types (2vHPV, 4vHPV, and 9vHPV), the percentage of these types tended to decrease with age (Table 2).

**Table 2:** Prevalence of high‐risk HPV based on HPV vaccine types, IARC classification, and number of high‐risk HPV infections per age group.

HPV	30–34 *N* = 435 *n* (%, 95% IC)	35–39 *N* = 235 *n* (%, 95% IC)	40–44 *N* = 150 *n* (%, 95% IC)	45–49 *N* = 95 *n* (%, 95% IC)	50–54 *N* = 137 *n* (%, 95% IC)	55–63 *N* = 148 *n* (%, 95% IC)
HPV vaccine types
2vHPV	202 (46%, 42–51)	105 (45%, 38–51)	57 (38%, 30–46)	37 (39%, 29–50)	41 (30%, 23–38)	37 (25%, 18–33)
4vHPV	209 (48%, 43–53)	109 (46%, 40–53)	62 (41%, 33–50)	38 (40%, 30–51)	49 (36%, 28–44)	40 (27%, 20–35)
9vHPV	351 (81%, 77–84)	182 (77%, 71–83)	112 (75%, 67–81)	70 (74%, 63–82)	92 (67%, 59–75)	98 (66%, 58–74)
IARC classification						
HPV16	175 40%, 36–45)	84 (36%, 30–42)	46 (31%, 24–39)	26 (27%, 19–38)	32 (23%, 17–32)	31 (21%, 15–29)
HPV18/45	47 (11%, 8.1–14)	32 (14%, 9.6–19)	18 (12%, 7.5–19)	18 (19%, 12– 29)	18 (13%, 8.2–20)	17 (11%, 7.0–18)
HPV33/58/31/52/35	129 (30%, 25–34)	67 (29%, 23–35)	46 (31%, 24–39)	28 (29%, 21–40)	38 (28%, 21–36)	52 (35%, 28–43)
HPV59/39/68/51/56	60 (14%, 11–17)	42 (18%, 13–24)	29 (19%, 14–27)	17 (18%, 11–27)	31 (23%, 16–31)	33 (22%, 16–30)
Negative for hrHPV	24 (5.5%, 3.6–8.2)	10 (4.3%, 2.2–7.9)	11 (7.3%, 3.9–13)	6 (6.3%, 2.6–14)	18 (13%, 8.2–20)	15 (10%, 6.0–16)
Number of hrHPV types						
0	24 (5.5%, 3.6–8.2)	10 (4.3%, 2.2–7.9)	11 (7.3%, 3.9–13)	6 (6.3%, 2.6–14)	18 (13%, 8.2–20)	15 (10%, 6.0–16)
1	261 (60%, 55–65)	154 (66%, 59–72)	100 (67%, 58–74)	66 (69%, 59–78)	83 (61%, 52–69)	95 (64%, 56–72)
2	92 (21%, 17–25)	53 (23%, 17–29)	29 (19%, 14–27)	17 (18%, 11–27)	25 (18%, 12–26)	20 (14%, 8.6–20)
≥ 3	58 (13%, 10–17)	18 (7.7%, 4.7–12)	10 (6.7%, 3.4–12)	6 (6.3%, 2.6–14)	11 (8.0%, 4.3–14)	18 (12%, 7.6–19)

### 3.5 Association Between With hrHPV Vaccine Types, Age, and Histologic Diagnosis

When we performed logistic regression to evaluate the association between 9vHPV types, age and histologic diagnosis (Table 3), we found that in a model with only age, for each additional year of age, the risk of having 9vHPV types decreased (OR: 0.97; 95% CI: −0.95,0.98). In a model with histological diagnosis (< CIN3 vs. CIN3 + ) women with CIN3+ were more likely to have the 9vHPV types compared to those < CIN3 (OR: 7.37; 95%CI: 4.71,12.19). In a model combing age and histological diagnosis, the association between CIN3+ and 9vHPV types remained (OR: 6.85; 95% CI: 4.36, 11.37).

**Table 3:** Association (OR) between high‐risk types included in the 9vHPV, age, and histologic diagnosis (logistic regression).

Model	OR	95% confidence interval	*p* value
Model 1 (hr‐*9vHPV ~ age)*	0.97	(0.95, 0.98)	< 0.001
Model 2 (hr‐9vHPV ~ CIN3 + )	7.37	(4.71, 12.19)	< 0.001
Model 3 (hr‐9vHPV ~ CIN3 + , adjusted by age)	6.85	(4.36, 11.37)	< 0.001

*Note:* Model 1: Age (a numeric variable) was the only independent variable. Model 2: Histologic diagnosis (< CIN3 vs. CIN3+) was the only independent variable. Model 3: Both age and histologic diagnosis were included as independent variables.

## 4 Discussion

This study provides an updated overview of HPV genotype prevalence among 1200 hrHPV‐positive, non‐vaccinated women aged 30–63 years old who participated in the Dutch population‐based cervical cancer screening program using a self‐sampler device. Our findings reveal that in the total cohort, at least one of any 32 HPV genotypes is identified in 98.8% of the samples and hrHPV is identified in 93% of the samples. HPV16, HPV31 and HPV52 are the most detected genotypes across the population. HPV16 is predominant in CIN3 and cancer cases, with prevalence rates of 66% and 85%, respectively, compared to 20% in ≤ CIN2. Additionally, the prevalence of HPV16 was around 40% in women aged 30–34 and decreases with age to 20% in women aged 55–63. This decline of HPV16 with age is also reflected in a lower HPV16 prevalence in cervical cancer with age, as reported in a systematic review from Hammer at al. [17] Moreover, over half of the women have multiple infections, although specific co‐infection patterns appear in very low frequencies Overall, ~40% of the samples have HPV types included in the 2vHPV/4vHPV vaccines and 74% have HPV types included in the 9vHPV vaccine.

Our study is consistent with previous studies conducted in Europe and among Dutch women at screening age, showing that HPV16 is the most prevalent genotype in both cervical and vaginal lesions [6, 13, 18]. Moreover, our findings reveal a progressive increase in HPV16 prevalence with the severity of the underlying histological diagnosis and a decreasing trend with the increase of age, which is also consistent with a previous report on Dutch women eligible for screening [13]. Our study also supports earlier findings that group d (HPV39/51/59/56/68), as classified by the IARC, is rarely found in high‐grade lesions and cancer. Therefore, genotypes HPV39, 51, 59, 56, and 68 may be less effective in predicting cervical cancer risk and may have limited utility when detected individually within primary screening [11]. More recently, HPV68 has been suggested not to be considered as a high‐risk genotype [19]. These findings highlight the need of region‐specific genotype selection in extended genotype HPV testing in the context of cervical cancer screening, particularly for non‐vaccinated cohorts, given that women with these genotypes may require a different screening algorithm, likely focusing on follow‐ups rather than colposcopy referral [11].

Although the 2vHPV vaccine remains in use in the Netherlands, our study suggests that adopting the nonavalent vaccine could offer substantial preventive benefits, potentially reducing CIN3+ cases by 90% to 100%, consistent with previous research finding [13, 20, 21].

For this study, we chose the INNO‐LiPA assay despite its unsuitability for PBS screening settings. This test has a high analytical sensitivity and is capable to identify 32 individual HPV genotypes. A feature not provided by most of the currently approved clinical tests, which typically indicates only some hrHPV types (HPV16 and 18), but mainly positivity without specifying other HPV types [7, 16]. The analytical sensitivity of INNO‐LiPA makes it a recommended choice for epidemiological studies investigating the prevalence and distribution of HPV types [16]. Using this assay, we identify 84 samples that tested negative for the 13 hrHPV types (79% were ≤ CIN1), although all included samples were hrHPV‐positive according to Cobas 4800 clinical hrHPV assay as part of the Dutch PBS program. Out of these 84 samples, 44 (52%) were positive for HPV66, a genotype dropped as a high‐risk type since 2009 due to insufficient evidence of its association with cervical cancer, but still part of the non‐HPV16/18 hrHPV group in the Cobas 4800 clinical hrHPV assay [7, 11]. Additionally, 8 samples (1× HPV66 and 7× noncarcinogenic types) showed very weak staining in the INNO‐LiPA assay indicating a very low viral load and considered as HPV‐negative in our study but still detectable in the Cobas 4800 assay. Among the remaining 32 samples that all showed sufficient human DNA input, 15 samples were negative for all HPV types and 17 were positive for low‐risk HPV types only in the INNO‐LiPA assay. Retesting all 32 samples with the Cobas 4800 clinical hrHPV assay yielded 12 valid HPV‐negative results, further suggesting a low viral load in these samples. We used 0.4 mL of each sample for DNA extraction for the Cobas 4800 HPV test and 0.3 mL for the INNO‐LiPA assay. We cannot exclude that this variation resulting in a different DNA yield, could affect the sensitivity of HPV detection. With our data, we cannot ensure whether these discrepancies are false positives, cross‐reactions with non‐hrHPV types or due to low viral load. Overall, these 12 of the 1200 samples is quite low compared to studies analyzing inter‐ and intra‐lab reproducibility with discordance of approximately 5% [22, 23], while when different HPV assays are examined discordance rates increase ranging from 31% to 52% [24, 25].

This study benefits from using self‐samples taken during the regular Dutch cervical cancer screening program and employing a highly sensitive analytic test. However, the study has some limitations. We selected self‐sampled material of women living in the North of the Netherlands, and the observed distribution might not be representative to other regions outside of the Netherlands. For instance, besides HPV16, HPV45 is more prevalent in Africa, while HPV52/58 are more predominant in China, therefore our observations for vaccination purpose are suitable especially for the Western‐European region [6, 11, 26].

In this study, we included samples from non‐vaccinated women. To monitor vaccine effectiveness, it is also important to analyze hrHPV genotypes in vaccinated women as it can be expected that the distribution of hrHPV genotypes in vaccinated cohorts will be different. For instance, in the United States, the most prevalent genotypes in vaccinated cohorts are HPV51, 52 and 39 [27]. In Costa Rica, a clinical trial reported that among CIN3+ cases from a cohort of girls vaccinated with the 2vHPV vaccine, 49% were attributed to HPV52 or 58, 41% were linked to carcinogenic types not covered by the nonavalent vaccine, 7% could not be attributed to a known carcinogenic type, and 2% had an unknown causal type [28]. In a Finnish cohort vaccinated with a 2vHPV, types HPV52, 59, 51, 58, and 33 being the most prevalent among high‐grade lesions [29]. Although this paper focuses on cervical screenings and cervical lesions, it is crucial to recognize that the benefits of the HPV vaccine extend beyond cervical cancer [30]. HPV is responsible for approximately 30% of all cancers caused by infectious agents, including anogenital cancers (such as vulvar, vaginal, penile, and anal cancers), as well as head and neck cancers (including tonsillar and pharyngeal cancers, and respiratory papillomatosis), and esophageal carcinomas [30]. Therefore, a reduction in the incidence of these cancers is also expected [30]. In conclusion, this study provides an updated view of HPV genotype prevalence among hrHPV‐positive women attending the Dutch cervical cancer screening program. HPV16, HPV31, and HPV52 were the most prevalent genotypes, with HPV16 being notably common in CIN3+ lesions and in younger women. We identify at least 32 different HPV genotypes in the Dutch population, with 30% of cases showing co‐infections with another hrHPV genotype. This distribution underscores the potential benefits of extended genotyping for refining management strategies for hrHPV‐positive women. Finally, the 9vHPV vaccine, which targets many of these prevalent high‐risk types, shows promise in reducing not only CIN3+ cases but also precancerous lesions like CIN1 and CIN2, highlighting its significant potential to improve cervical cancer and other HPV‐related cancer.

## Author Contributions

**Kelly M. Castañeda:** conceptualization, investigation, data curation, methodology, formal analysis, writing – original draft. **Jolien de Waard:** investigation, data curation, writing – review and editing. **Lorian Slagter‐Menkema:** data curation, investigation, writing – review and editing. **Mirjam Mastik:** investigation, writing – review and editing. **Floris Adriaan Vuijk:** project administration, conceptualization, writing – review and editing. **Rudolf Lambertus Maria:** writing – review and editing. **Geertruida Hendrika de Bock:** methodology, supervision, writing, review, and editing. **Gijsbertha Barendina Alida Wisman:** conceptualization, methodology, resources, supervision, writing, review, and editing. **Ed Schuuring:** conceptualization, methodology, resources, supervision, writing, review, and editing. The work reported in the paper was performed by the authors, unless clearly specified in the text. All authors reviewed and approved the final version of the paper.

## Ethics Statement

Consent for use of the biobank was obtained according to the local regulations and those from the National Institute for Health and the Environment (RIVM).

## Conflicts of Interest

E.S. is a member of the scientific advisory board of Roche, Hologic Inc, QCMD, and CC Diagnostics; received grants from CC Diagnostics, Abbott Molecular Inc. and MSD B.V.; and received travel reimbursements from Roche, Abbott Molecular Inc., Hologic Inc., and QCMD (all honoraria paid to UMCG account). G.B. A.W. is a member of the scientific advisory board of CC Diagnostics and Novosanis; and received grants from CC Diagnostics, Abbott Molecular Inc., and MSD B.V. (all honoraria paid to UMCG account). F.A.V. is an employee of MSD B.V. (The Netherlands) that funded this study. R.L.M.B. received speakers' fees from MSD B.V. The other authors declare no conflicts of interest.

## Supporting information

Supporting tables and figure.

## Data Availability

The data that support the findings of this study are available on request from the corresponding author. The data are not publicly available due to privacy or ethical restrictions.
