# Supplementary material for: Distribution of Human Papillomavirus Genotypes in Real‐World Cervical Self‐Collected Scrapings From the Dutch Cervical Cancer Screening Program
Source: J Med Virol. 2025 Jul 2;97(7):e70461. doi: 10.1002/jmv.70461 (PMC12216794; doi:10.1002/jmv.70461)
Supplement: Supplementary file 1 — Supporting tables and figure. [file JMV-97-e70461-s001.docx]

**Supplementary tables and figure**

***Supplementary table 1. Distribution of age group according to histology diagnosis***

| **Age group** | **Overall**  N = 1,200 n (%) | **NILM**  N = 400 n (%) | **CIN0/1**  N = 315 n (%) | **CIN2**  N = 164 n (%) | **CIN3/AIS**  N = 308 n (%) | **Cancer**  N = 13 n (%) |
| --- | --- | --- | --- | --- | --- | --- |
| 30-34 | 435 (36%) | 124 (31%) | 97 (31%) | 67 (41%) | 142 (46%) | 5 (38%) |
| 35-39 | 235 (20%) | 65 (16%) | 64 (20%) | 36 (22%) | 68 (22%) | 2 (15%) |
| 40-44 | 150 (13%) | 47 (12%) | 43 (14%) | 21 (13%) | 39 (13%) | 0 (0%) |
| 45-49 | 95 (7.9%) | 34 (8.5%) | 25 (7.9%) | 6 (3.7%) | 28 (9.1%) | 2 (15%) |
| 50-54 | 137 (11%) | 56 (14%) | 46 (15%) | 17 (10%) | 17 (5.5%) | 1 (7.7%) |
| 55-63 | 148 (12%) | 74 (19%) | 40 (13%) | 17 (10%) | 14 (4.5%) | 3 (23%) |

***Supplementary table 2. Distribution of HPV genotypes according to histology diagnosis***

| **Type** | **HPV** | **Overall**  N = 1,200 n (%) | **NILM**  N = 400 n (%) | **CIN0/1**  N = 315 n (%) | **CIN2**  N = 164 n (%) | **CIN3/AIS**  N = 308 n (%) | **Cancer**  N = 13 n (%) |
| --- | --- | --- | --- | --- | --- | --- | --- |
| hrHPV | HPV16 | 394 (33%) | 58 (15%) | 59 (19%) | 62 (38%) | 204 (66%) | 11 (85%) |
|  | HPV18 | 107 (8.9%) | 37 (9.3%) | 27 (8.6%) | 15 (9.1%) | 26 (8.4%) | 2 (15%) |
|  | HPV31 | 216 (18%) | 72 (18%) | 57 (18%) | 37 (23%) | 49 (16%) | 1 (7.7%) |
|  | HPV33 | 81 (6.8%) | 18 (4.5%) | 24 (7.6%) | 11 (6.7%) | 27 (8.8%) | 1 (7.7%) |
|  | HPV35 | 41 (3.4%) | 14 (3.5%) | 13 (4.1%) | 6 (3.7%) | 8 (2.6%) | 0 (0%) |
|  | HPV39 | 67 (5.6%) | 26 (6.5%) | 22 (7.0%) | 8 (4.9%) | 11 (3.6%) | 0 (0%) |
|  | HPV45 | 77 (6.4%) | 33 (8.3%) | 22 (7.0%) | 12 (7.3%) | 10 (3.2%) | 0 (0%) |
|  | HPV51 | 126 (11%) | 45 (11%) | 52 (17%) | 10 (6.1%) | 18 (5.8%) | 1 (7.7%) |
|  | HPV52 | 199 (17%) | 48 (12%) | 63 (20%) | 37 (23%) | 50 (16%) | 1 (7.7%) |
|  | HPV56 | 85 (7.1%) | 39 (9.8%) | 32 (10%) | 8 (4.9%) | 6 (1.9%) | 0 (0%) |
|  | HPV58 | 88 (7.3%) | 25 (6.3%) | 22 (7.0%) | 21 (13%) | 20 (6.5%) | 0 (0%) |
|  | HPV59 | 91 (7.6%) | 39 (9.8%) | 32 (10%) | 8 (4.9%) | 12 (3.9%) | 0 (0%) |
|  | HPV68 | 60 (5.0%) | 24 (6.0%) | 19 (6.0%) | 8 (4.9%) | 9 (2.9%) | 0 (0%) |
| phrHPV | HPV26 | 4 (0.3%) | 0 (0%) | 1 (0.3%) | 3 (1.8%) | 0 (0%) | 0 (0%) |
|  | HPV53 | 100 (8.3%) | 38 (9.5%) | 37 (12%) | 11 (6.7%) | 14 (4.5%) | 0 (0%) |
|  | HPV66 | 125 (10%) | 54 (14%) | 38 (12%) | 17 (10%) | 16 (5.2%) | 0 (0%) |
|  | HPV70 | 50 (4.2%) | 18 (4.5%) | 16 (5.1%) | 5 (3.0%) | 11 (3.6%) | 0 (0%) |
|  | HPV73 | 40 (3.3%) | 9 (2.3%) | 19 (6.0%) | 5 (3.0%) | 7 (2.3%) | 0 (0%) |
|  | HPV82 | 40 (3.3%) | 7 (1.8%) | 13 (4.1%) | 5 (3.0%) | 15 (4.9%) | 0 (0%) |
| lrHPV | HPV06 | 35 (2.9%) | 9 (2.3%) | 14 (4.4%) | 4 (2.4%) | 7 (2.3%) | 1 (7.7%) |
|  | HPV11 | 13 (1.1%) | 2 (0.5%) | 9 (2.9%) | 0 (0%) | 2 (0.6%) | 0 (0%) |
|  | HPV40 | 10 (0.8%) | 4 (1.0%) | 6 (1.9%) | 0 (0%) | 0 (0%) | 0 (0%) |
|  | HPV42 | 3 (0.3%) | 1 (0.3%) | 0 (0%) | 1 (0.6%) | 1 (0.3%) | 0 (0%) |
|  | HPV43 | 7 (0.6%) | 5 (1.3%) | 1 (0.3%) | 1 (0.6%) | 0 (0%) | 0 (0%) |
|  | HPV44 | 45 (3.8%) | 21 (5.3%) | 11 (3.5%) | 4 (2.4%) | 8 (2.6%) | 1 (7.7%) |
|  | HPV54 | 32 (2.7%) | 15 (3.8%) | 11 (3.5%) | 2 (1.2%) | 4 (1.3%) | 0 (0%) |
|  | HPV61 | 64 (5.3%) | 21 (5.3%) | 21 (6.7%) | 11 (6.7%) | 10 (3.2%) | 1 (7.7%) |
|  | HPV81 | 9 (0.8%) | 3 (0.8%) | 1 (0.3%) | 3 (1.8%) | 2 (0.6%) | 0 (0%) |
| Unclassified | HPV67 | 9 (0.8%) | 2 (0.5%) | 4 (1.3%) | 2 (1.2%) | 1 (0.3%) | 0 (0%) |
|  | HPV62 | 49 (4.1%) | 23 (5.8%) | 12 (3.8%) | 3 (1.8%) | 11 (3.6%) | 0 (0%) |
|  | HPV83 | 31 (2.6%) | 8 (2.0%) | 14 (4.4%) | 4 (2.4%) | 5 (1.6%) | 0 (0%) |
|  | HPV89 | 43 (3.6%) | 20 (5.0%) | 13 (4.1%) | 2 (1.2%) | 8 (2.6%) | 0 (0%) |
| HPV Negative | NA | 15 (1.3%) | 7 (1.8%) | 4 (1.3%) | 3 (1.8%) | 1 (0.3%) | 0 (0%) |

hrHPV: high-risk HPV genotypes

phrHP: possible high-risk HPV genotypes

lrHPV: low-risk HPV genotypes

The columns do not add up to 100% as a result of coinfections***Supplementary table 3. Distribution of HPV genotypes according to age groups***

| **Type** | **HPV** | | **Overall  N = 1,200 n (%)** | **30-34  N = 435 n (%)** | **35-39  N = 235 n (%)** | **40-44  N = 150 n (%)** | **45-49 N = 95 n (%)** | **50-54 N = 137 n (%)** | **55-63 N = 148 n (%)** |
| --- | --- | --- | --- | --- | --- | --- | --- | --- | --- |
| hrHPV | HPV16 | 394 (33%) | | 175 (40%) | 84 (36%) | 46 (31%) | 26 (27%) | 32 (23%) | 31 (21%) |
|  | HPV18 | 107 (8.9%) | | 39 (9.0%) | 24 (10%) | 12 (8.0%) | 14 (15%) | 10 (7.3%) | 8 (5.4%) |
|  | HPV31 | 216 (18%) | | 88 (20%) | 44 (19%) | 27 (18%) | 16 (17%) | 20 (15%) | 21 (14%) |
|  | HPV33 | 81 (6.8%) | | 33 (7.6%) | 12 (5.1%) | 10 (6.7%) | 5 (5.3%) | 7 (5.1%) | 14 (9.5%) |
|  | HPV35 | 41 (3.4%) | | 12 (2.8%) | 9 (3.8%) | 3 (2.0%) | 4 (4.2%) | 7 (5.1%) | 6 (4.1%) |
|  | HPV39 | 67 (5.6%) | | 25 (5.7%) | 14 (6.0%) | 8 (5.3%) | 4 (4.2%) | 5 (3.6%) | 11 (7.4%) |
|  | HPV45 | 77 (6.4%) | | 28 (6.4%) | 14 (6.0%) | 7 (4.7%) | 7 (7.4%) | 9 (6.6%) | 12 (8.1%) |
|  | HPV51 | 126 (11%) | | 44 (10%) | 22 (9.4%) | 15 (10%) | 10 (11%) | 16 (12%) | 19 (13%) |
|  | HPV52 | 199 (17%) | | 77 (18%) | 40 (17%) | 28 (19%) | 11 (12%) | 19 (14%) | 24 (16%) |
|  | HPV56 | 85 (7.1%) | | 26 (6.0%) | 10 (4.3%) | 11 (7.3%) | 5 (5.3%) | 18 (13%) | 15 (10%) |
|  | HPV58 | 88 (7.3%) | | 38 (8.7%) | 13 (5.5%) | 8 (5.3%) | 7 (7.4%) | 8 (5.8%) | 14 (9.5%) |
|  | HPV59 | 91 (7.6%) | | 31 (7.1%) | 21 (8.9%) | 11 (7.3%) | 7 (7.4%) | 10 (7.3%) | 11 (7.4%) |
|  | HPV68 | 60 (5.0%) | | 24 (5.5%) | 13 (5.5%) | 4 (2.7%) | 2 (2.1%) | 8 (5.8%) | 9 (6.1%) |
| phrHPV | HPV26 | 4 (0.3%) | | 2 (0.5%) | 1 (0.4%) | 0 (0%) | 0 (0%) | 0 (0%) | 1 (0.7%) |
|  | HPV53 | 100 (8.3%) | | 41 (9.4%) | 17 (7.2%) | 9 (6.0%) | 7 (7.4%) | 13 (9.5%) | 13 (8.8%) |
|  | HPV66 | 126 (11%) | | 41 (9.4%) | 23 (9.8%) | 14 (9.3%) | 4 (4.2%) | 26 (19%) | 18 (12%) |
|  | HPV70 | 50 (4.2%) | | 22 (5.1%) | 7 (3.0%) | 3 (2.0%) | 4 (4.2%) | 5 (3.6%) | 9 (6.1%) |
|  | HPV73 | 40 (3.3%) | | 16 (3.7%) | 6 (2.6%) | 4 (2.7%) | 5 (5.3%) | 2 (1.5%) | 7 (4.7%) |
|  | HPV82 | 40 (3.3%) | | 16 (3.7%) | 6 (2.6%) | 10 (6.7%) | 1 (1.1%) | 4 (2.9%) | 3 (2.0%) |
| lrHPV | HPV06 | 35 (2.9%) | | 8 (1.8%) | 7 (3.0%) | 7 (4.7%) | 2 (2.1%) | 8 (5.8%) | 3 (2.0%) |
|  | HPV11 | 13 (1.1%) | | 6 (1.4%) | 2 (0.9%) | 1 (0.7%) | 1 (1.1%) | 2 (1.5%) | 1 (0.7%) |
|  | HPV40 | 10 (0.8%) | | 6 (1.4%) | 2 (0.9%) | 1 (0.7%) | 0 (0%) | 0 (0%) | 1 (0.7%) |
|  | HPV42 | 3 (0.3%) | | 1 (0.2%) | 0 (0%) | 0 (0%) | 1 (1.1%) | 0 (0%) | 1 (0.7%) |
|  | HPV43 | 7 (0.6%) | | 2 (0.5%) | 0 (0%) | 1 (0.7%) | 0 (0%) | 3 (2.2%) | 1 (0.7%) |
|  | HPV44 | 45 (3.8%) | | 10 (2.3%) | 8 (3.4%) | 4 (2.7%) | 5 (5.3%) | 7 (5.1%) | 11 (7.4%) |
|  | HPV54 | 32 (2.7%) | | 15 (3.4%) | 9 (3.8%) | 3 (2.0%) | 1 (1.1%) | 3 (2.2%) | 1 (0.7%) |
|  | HPV61 | 64 (5.3%) | | 21 (4.8%) | 12 (5.1%) | 7 (4.7%) | 6 (6.3%) | 17 (12%) | 1 (0.7%) |
|  | HPV81 | 9 (0.8%) | | 3 (0.7%) | 3 (1.3%) | 1 (0.7%) | 0 (0%) | 1 (0.7%) | 1 (0.7%) |
| Unclassified | HPV67 | 9 (0.8%) | | 0 (0%) | 2 (0.9%) | 2 (1.3%) | 1 (1.1%) | 3 (2.2%) | 1 (0.7%) |
|  | HPV62 | 49 (4.1%) | | 7 (1.6%) | 10 (4.3%) | 8 (5.3%) | 6 (6.3%) | 6 (4.4%) | 12 (8.1%) |
|  | HPV83 | 31 (2.6%) | | 9 (2.1%) | 3 (1.3%) | 6 (4.0%) | 1 (1.1%) | 4 (2.9%) | 8 (5.4%) |
|  | HPV89 | 43 (3.6%) | | 14 (3.2%) | 7 (3.0%) | 9 (6.0%) | 1 (1.1%) | 7 (5.1%) | 5 (3.4%) |
| HPV Negative |  | 15 (1.3%) | | 8 (1.8%) | 0 (0%) | 1 (0.7%) | 1 (1.1%) | 2 (1.5%) | 3 (2.0%) |

hrHPV: high-risk HPV genotypes

phrHP: possible high-risk HPV genotypes

lrHPV: low-risk HPV genotypes

The columns do not add up to 100% as a result of coinfections


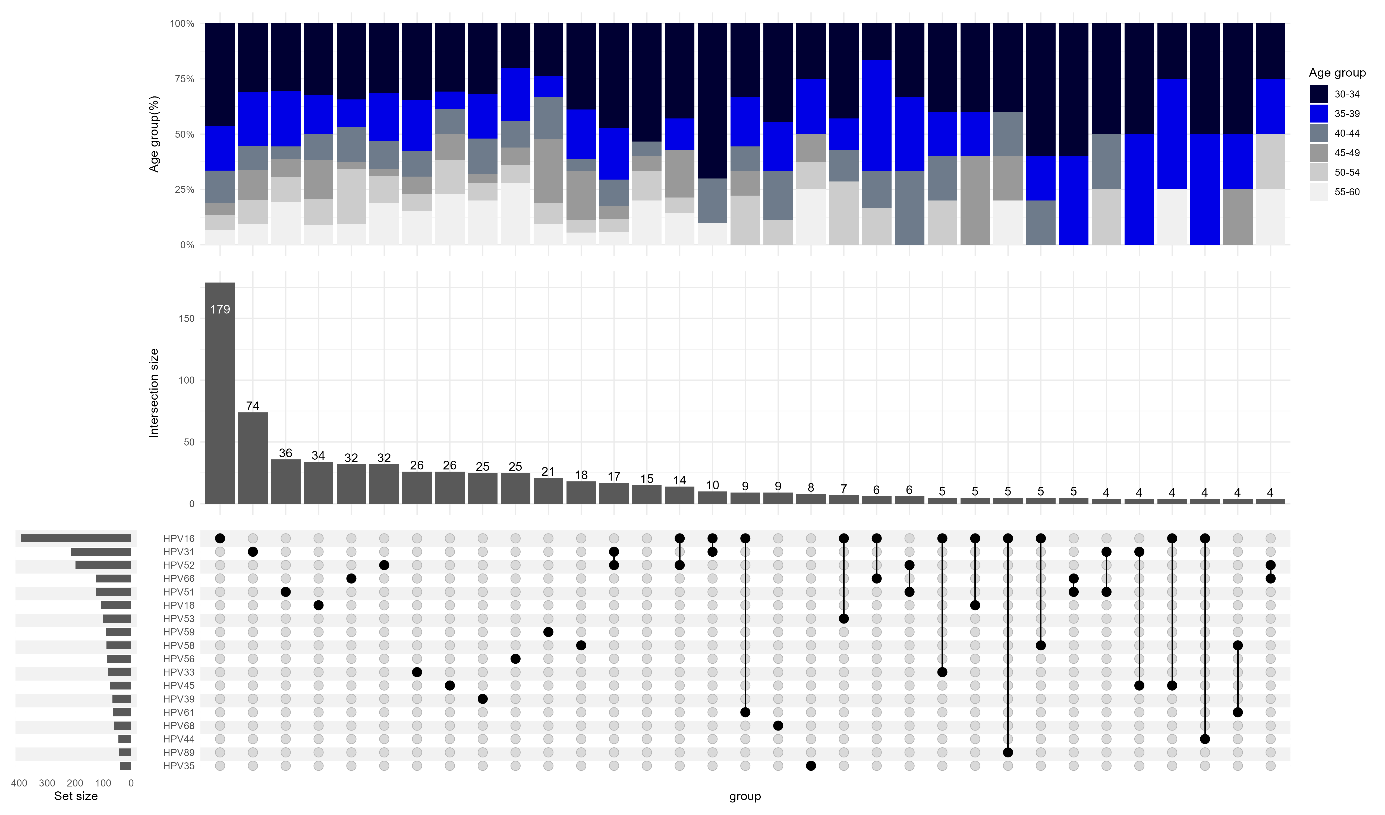


**Supplementary Figure 1.** Upset plot showing the frequencies of HPV genotypes (single and multiple infections) in Set size, and the pattern of HPV infections in intersection size. Each dot in the matrix represents positivity for the respective HPV in the row. When two dots are connected by a line, it denotes a coinfection. The stacked bars at the top indicate the percentages of age group per patterns of HPV infections. Only patterns of HPV infections occurring in four or more participants are presented. Ranking is based on frequency.
